# Analysis of risk factors for carbapenem-resistant *Klebsiella pneumoniae* bloodstream infections in children: a case-control study

**DOI:** 10.1186/s12866-026-05342-8

**Published:** 2026-07-03

**Authors:** Jing Jia, Hailing Shi, Panpan Fang, Juanjuan Zhou, Lu Xu, Kaijie Gao, Haizhen Xie, Bing Yang, Mingfa Guo

**Affiliations:** 1 Department of Clinical Laboratory, Children’s Hospital Affiliated to Zhengzhou University, Zhengzhou Key Laboratory of Children’s Infection and Immunity, Zhengzhou, China; 2 Department of Hematology and Oncology, Children’s Hospital Affiliated to Zhengzhou University Key Laboratory of Hematology, Zhengzhou, Henan 450000 China

**Keywords:** Children, Carbapenem-resistant *Klebsiella pneumoniae*, Bloodstream infection

## Abstract

**Objective:**

To analyze the risk factors for carbapenem-resistant *Klebsiella pneumoniae* bloodstream infection (CRKP-BSI) in children.

**Methods:**

A total of 235 pediatric patients with *Klebsiella pneumoniae* bloodstream infection (KPN-BSI) admitted to our hospital from January 2015 to December 2025 were collected. According to the CLSI criteria, infections in which carbapenem- resistant *Klebsiella pneumoniae* was isolated from blood culture were defined as CRKP-BSI (*n*=110), and those in which carbapenem-susceptible *Klebsiella pneumoniae* was isolated were defined as CSKP-BSI (*n*=125). We compared clinical profiles of the two groups with the Mann-Whitney U test and chi-square test, and binary logistic regression was used to identify independent risk factors for CRKP-BSI.

**Results:**

Children with CRKP-BSI were mainly distributed in the Premature Infant Department (49 cases, 44.60 %) and intensive care unit (36 cases, 32.70 %). Analysis of clinical characteristics showed that patient age, underlying diseases, antibiotic use duration ≥ 14 days, mechanical ventilation, surgical procedures during hospitalization, and hospital stay > 5 days were significantly associated with CRKP-BSI (all *P* < 0.05). Binary logistic regression multivariate analysis demonstrated that the mechanical ventilation (*β* = 2.896, OR = 18.106), surgical procedures during hospitalization (*β* = 1.116, *OR* = 3.052), and hospital stay > 5 days (*β* = 0.957, OR = 2.603) were independently associated with CRKP-BSI.

**Conclusion:**

It is concluded that mechanical ventilation, surgical interventions and prolonged hospitalization independently contribute to the development of CRKP-BSI in children. Enhanced monitoring and precise infection control strategies are required for vulnerable children to prevent CRKP-BSI.

**Supplementary Information:**

The online version contains supplementary material available at https://doi.org/10.1186/s12866-026-05342-8.

## Introduction

*Klebsiella pneumoniae* (KP) is a Gram-negative bacillus and a common pathogenic bacterium with strong virulence that frequently causes severe infections in clinical practice. Bloodstream infection (BSI) is one of the critical conditions in pediatric patients, significantly prolonging hospital stays, increasing medical costs, and even endangering lives. Studies have shown that KP-associated BSI has a detection rate of 85.60%, with a mortality rate as high as 24.30% in pediatric patients, posing a serious threat to children’s health [1].

With the widespread use of broad-spectrum antibiotics, carbapenem-resistant *Klebsiella pneumoniae*(CRKP) has emerged as a global priority pathogen for surveillance and infection control. CRKP can cause septic shock and multiple organ failure, with high fatality rates, posing a serious threat to human health [2]. In children, who represent a particularly vulnerable population due to their immunologically immature systems and frequent coexistence of underlying comorbidities. Coupled with the restricted use of multiple antibiotics, clinical anti-infection treatment faces significant challenges. The present study was designed to analyze the clinical characteristics of CRKP-BSI in children and to determine the independent risk factors using binary logistic regression, with the aim of providing evidence-based guidance for the early identification of high-risk children and the development of targeted prevention, control, and anti-infective treatment strategies.

## Methods

### Study design and patients

This study collected data from 235 pediatric patients with KP bloodstream infections at Henan Children’s Hospital from January 2015 to December 2025. After excluding duplicate isolates from the same patient, 110 children with CRKP-BSI were assigned to the CRKP-BSI group, and 125 children with CSKP-BSI were assigned to the CSKP-BSI group. Clinical data for both groups were collected, including gender, age, underlying diseases, hospital departments, invasive procedures, and laboratory indicators. Inclusion criteria [3]: (1) clinically confirmed KP-BSI; (2) comprehensive clinical documentation is avaiable; (3) meeting the U.S. Centers for Disease Control and Prevention (CDC) diagnostic criteria for BSI: pathogens that are not common skin colonizers detected in blood cultures can confirm bloodstream infection with a single positive result, provided the isolated isolate is unrelated to infections at other body sites; for common skin commensals such as coagulase-negative staphylococci, diagnosis requires at least two sets of blood cultures collected from different sites on separate occasions to yield the same isolate; together with clinical signs of infection such as fever, chills, or hypotension, and exclusion of other primary infection foci.

### Ethics approval

This retrospective clinical study was approved by the Ethics Committee of Henan Children’s Hospital (Approval No.: 2022-K-L030). All clinical data were anonymized and de-identified prior to protect patient privacy. Informed consent was waived by the Ethics Committee due to the retrospective nature of the study. The study protocol complied with the ethical principles of the Declaration of Helsinki.

### Definition of clinical variables

Predictive factors examined in this study included age, underlying disease, invasive procedures, surgical classification and antimicrobial susceptibility phenotypes of isolates.

Age was recorded based on the actual age at admission. Underlying diseases referred to pre-existing comorbidities prior to hospitalization, including cardiovascular and cerebrovascular diseases, diabetes mellitus, renal dysfunction, hematological diseases and immunodeficiency.

Invasive procedures were represented by mechanical ventilation. Prolonged hospitalization was defined as a hospital stay of more than 5 days (duration from admission to positive blood culture). Long-term antibiotic use was defined as administration for no less than 14 days before blood culture yielded positive results.

According to the Measures for the Classification and Administration of Surgeries in Medical Institutions (National Health Commission General Office Document No.〔2022〕18), surgeries were divided into four grades: grade 1 (low risk, simple procedure, low technical difficulty), grade 2 (some risk, moderate complexity, some technical difficulty), grade 3 (higher risk, more complex, more difficult, greater resource consumption), and grade 4 (highest risk, complex procedure, high difficulty, high resource consumption).

Antimicrobial susceptibility and resistance of bacterial isolates were determined in accordance with the Clinical and Laboratory Standards Institute (CLSI) breakpoints: susceptible isolates can be effectively inhibited by antimicrobial agents at conventional therapeutic doses; resistant isolates cannot be suppressed by standard-dose antimicrobial therapy, typically leading to clinical treatment failure.

### Isolate identification and drug susceptibility test results

Bacterial identification was performed using matrix-assisted laser desorption ionization-time of flight mass spectrometry (MALDI-TOF MS; Bruker, Germany) with the corresponding target plates and reagent kits (Sigma, USA): The test isolates and quality control isolates were spotted onto the target plate: firstly, 1 µl of 70% formic acid was added and mixed thoroughly, followed by air-drying at room temperature; subsequently, 1 µl of matrix solution was applied and allowed to dry naturally; finally, the target plate was placed into the MALDI-TOF MS system for spectral acquisition and isolate identification.

Antimicrobial susceptibility testing was carried out using the VITEK 2-Compact automated microbiology system (bioMérieux, France): The test isolates and quality control isolates were adjusted to the standard McFarland turbidity and further diluted as required; after sample information was entered, antimicrobial susceptibility cards and bacterial suspension were loaded. The instrument automatically performed sample loading and filling, and determined the minimum inhibitory concentration (MIC) according to the preset growth parameters of the isolates. Susceptibility results were interpreted according to the current CLSI standards for the relevant testing year. ATCC 700,603 was used as the quality control isolate throughout.

### Instruments and reagents

The following instruments and reagents were used in this study: the VITEK 2-Compact automated microbial identification and susceptibility testing system with corresponding susceptibility cards (bioMérieux, France); the MALDI-TOF MS mass spectrometer with corresponding reagent kits and target plates (Bruker, Germany; Sigma, USA); culture media, including Columbia agar, chocolate agar, Mueller–Hinton agar, and Mueller–Hinton agar supplemented with 5% defibrinated sheep blood, as well as antibiotic susceptibility discs (Oxoid, UK); ceftriaxone and meropenem Etest strips (Autobio, Zhengzhou, China); and a Gram staining kit (rapid method, Zhuhai Beso Biotechnology, China).

### CRKP isolate confirmation

The selected CRKP isolates were re-identified using imipenem and meropenem Etest strips (Autobio, Zhengzhou, China), with results interpreted according to the CLSI breakpoints applicable in each respective year of testing.

### Laboratory indicators

At the time of blood culture collection, 4 ml of peripheral blood was obtained from each child: 2 mL was dispensed into an EDTA-K₂ anticoagulant tube for whole-blood analysis, and the remaining 2 mL was collected into a serum separator tube. White blood cell (WBC) count and C-reactive protein (CRP, latex agglutination method) levels were measured using a Mindray BC-7500 hematology analyzer. The serum separator tube was centrifuged at 3,500 rpm for 15 min to obtain serum, and procalcitonin (PCT) levels were measured using a Roche Cobas 801 analyzer.

### Statistical methods

Statistical analyses were performed using SPSS software (version 21.0, IBM). Continuous variables that were not normally distributed (e.g., age and laboratory indicators) were expressed as median with interquartile range [M (P₂₅, P₇₅)], and between-group comparisons were performed using the non-parametric Mann–Whitney U test. Categorical variables were described as numbers with percentages (%), and between-group differences were analyzed using the chi-square (χ²) tests.

This study used a retrospective, unmatched case-control design; the sample size was determined by all eligible consecutive cases identified during the study period. With a significance level of α = 0.05 and a medium effect size, the calculated statistical power (1 − β) was 0.968 and 0.952, respectively, both exceeding 0.8, indicating that the sample size was adequate to detect true between-group differences. To minimize selection bias, information bias, and confounding bias, the following measures were implemented: uniform inclusion and exclusion criteria; exclusion of duplicate isolates from the same patient; double data entry with cross-verification to ensure data quality; and binary logistic regression analysis to adjust for potential confounders in identifying independent risk factors for CRKP-BSI in children.Variables with a *p*-value < 0.05 in univariate analysis were entered into the multivariate logistic regression model using the enter method. A two-sided *p*-value < 0.05 was considered statistically significant.

## Result

### Demographic characteristics

**Fig. 1:**
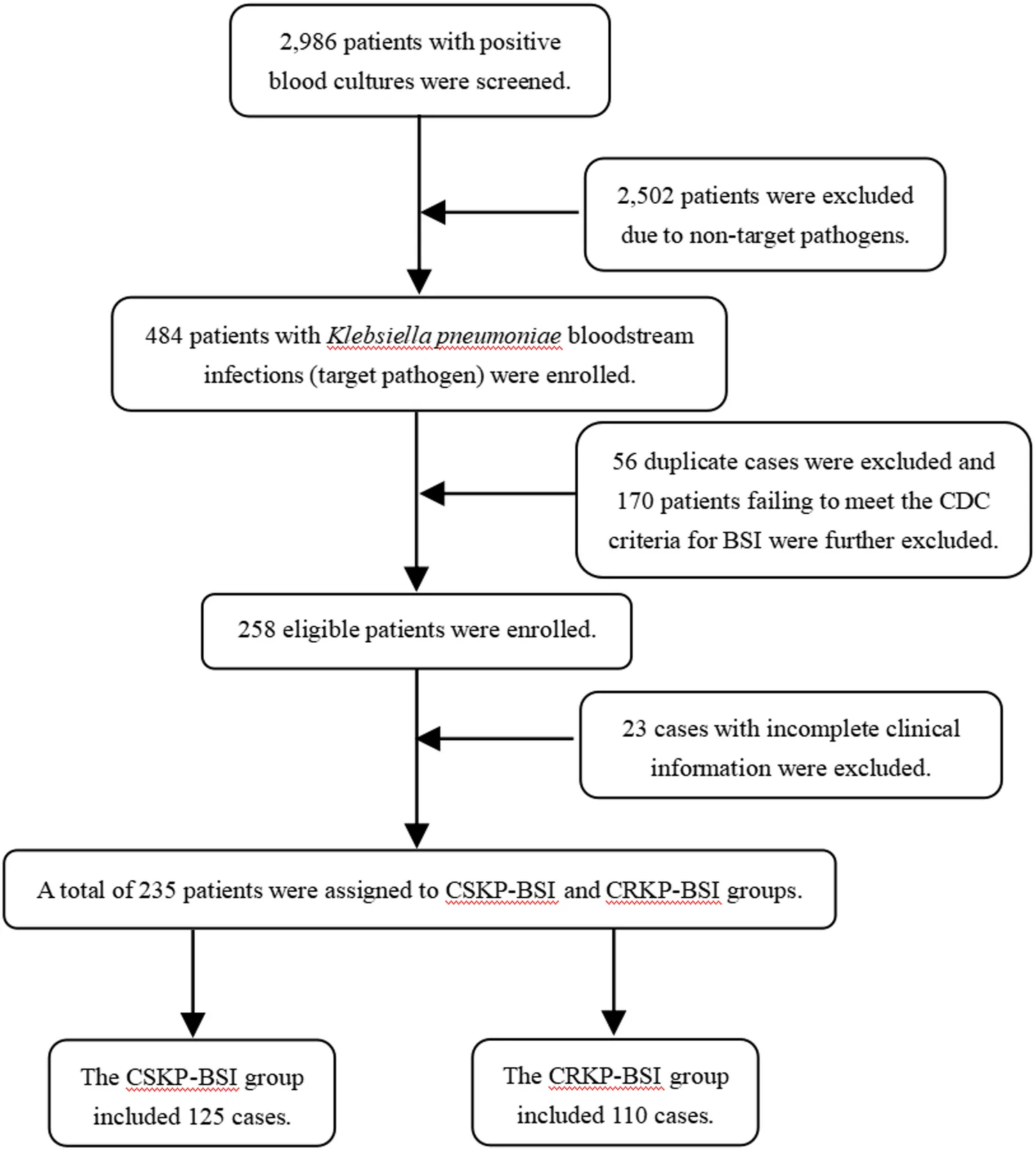
Flowchart of study recruitment

A total of 2,986 pediatric patients with positive blood cultures were initially screened. Of these, 2,502 patients were excluded due to non-target pathogens. After clinical data verification and diagnostic criteria review, 235 patients were finally included in the analysis. According to the study design, patients were divided into the CRKP-BSI group (110 cases, 46.81%) and the CSKP-BSI group (125 cases, 53.19%). The detailed exclusion process and participant screening flowchart are presented in Fig. 1.

### Departmental distribution

Patients in both the CRKP-BSI and CSKP-BSI groups were predominantly distributed in the Premature Infant Department and the Intensive Care Unit. However, significant differences in departmental distribution between the two groups were observed for the Premature Infant Department, Surgery Department, and Hematology-Oncology Department (all P < 0.05), as detailed in Table 1.

**Table 1:** Departmental distribution of CRKP-BSI and CSKP-BSI groups in pediatric patients

Department	CRKP-BSI (*n* = 110)	CSKP-BSI (*n* = 125)	χ^2^	*P*
Premature Infant Department	49(44.55)	18(14.40)	26.087	<0.001
Intensive Care Unit	36(32.73)	28(22.40)	3.149	0.076
Neonatology Department	10(9.09)	15(12.00)	0.521	0.470
Surgery Department	9(8.18)	26(20.80)	7.350	0.007
Hematology-Oncology Department	4(3.64)	30(24.00)	19.607	<0.001
Endocrinology Department	1(0.91)	0	-	-
Infant Department	1(0.91)	4(3.20)	-	-
Infectious Diseases Department	0	1(0.80)	-	-
Gastroenterology Department	0	1(0.80)	-	-
Nephrology-Rheumatology Department	0	2(1.60)	-	-

*Abbreviations*: *CRKP* carbapenem-resistant *Klebsiella pneumoniae*, *CSKP* carbapenem-susceptible *K. pneumoniae*, *BSI* bloodstream infection

Data are presented as *n* (%).The symbol “–” indicates that statistical comparison was not performed due to small cell counts (*n* < 3)

### Comparison of general clinical characteristics

A total of 235 pediatric patients were included in this study, comprising 143 males and 92 females. The majority of patients in both groups were neonates. Significant differences between the two groups were observed in age, history of underlying diseases, duration of antibiotic use ≥ 14 days, hospital stay > 5 days, mechanical ventilation, and surgical procedures (all P < 0.05), as detailed in Table 2.

**Table 2:** Clinical characteristics of pediatric patients with CRKP-BSI and CSKP-BSI

Clinical features	CRKP-BSI (*n* = 110)	CSKP-BSI (*n* = 125)	χ^2^/Z	*P*
Gender, *n *(%)				
Male	64(58.20)	79(63.20)	0.618	0.432
Female	46(41.80)	46(36.80)		
Age, *n *(%)				
≤ 28 days	78(70.91)	56(44.80)	19.459	0.001
≤ 1 y	24(21.82)	38(30.40)		
>1 y	8(7.27)	31(24.80)		
Underlying diseases, *n *(%)				
Digestive diseases	5(4.55)	19(15.20)	7.234	0.007
Congenital heart disease	9(8.18)	4(3.20)	2.799	0.096
Premature infants	66(60.00)	26(20.80)	34.741	0.001
Neoplastic diseases	3(2.73)	27(21.60)	18.714	0.001
Hospitalization data, *n *(%)				
Duration of antibiotic use ≥ 14 days	53(48.18)	37(29.60)	8.549	0.003
Duration of antibiotic use < 3 days	16(14.55)	43(34.40)	12.267	0.001
Immunosuppressive therapy	42(38.18)	51(40.80)	0.168	0.682
Hospital stay > 5 days	94(82.73)	65(52.00)	29.927	0.001
Mechanical ventilation	82(74.55)	28(22.40)	63.900	0.001
Surgical procedure	25^a^(22.73)	7^b^(5.60)	14.592	0.001
Outcome, *n *(%)				
Improvement	91(82.73)	111(88.80)	1.788	0.181
Death	19(17.27)	14(11.20)		
Laboratory indicators, median(IQR)				
WBC (×10^9^/L)	10.22(4.96,17.42)	9.60(3.85,16.03)	-1.141	0.254
Lymphocyte percentage (*n*%)	23.15(15.75,39.08)	27.35(13.53,49.93)	-1.598	0.110
Neutrophil percentage (*n*%)	68.40(50.6,78.25)	64.25(34.48,80.55)	-1.583	0.113
PLT (×10^9^/L)	104.00(34.50,207.25)	116.00(34.00,250.00)	-0.289	0.772
CRP (mg/L)	39.50(10.26,69.77)	27.81(2.63,74.21)	-0.880	0.379
PCT (ng/L)	3.83(0.907,35.13)	5.79(0.39,25.92)	-0.110	0.912
CRE (µmol/L)	34.60(26.18,56.03)	37.70(23.48,64.20)	-0.276	0.783
ALT (U/L)	27.25(16.05,61.58)	31.00(19.50,60.80)	-1.568	0.117

Data are presented as n(%) for categorical variables or median (interquartile range, IQR) for continuous variables. *p* values were calculated using the chi-square (χ²) test for categorical variables and the Mann–Whitney U test for continuous variables

*Abbreviations*: *CRKP-BSI* carbapenem-resistant *Klebsiella pneumoniae* bloodstream infection, *CSKP-BSI* carbapenem-susceptible *Klebsiella pneumoniae* bloodstream infection, *WBC* white blood cell, *PLT* platelet, *CRP* C-reactive protein, *CRE* creatinine, *ALT* alanine aminotransferase

^a^24 /25(96.0%) patients underwent grade 3 or 4 surgeries. ^b^7/7 (100%) patients underwent grade 3 or 4 surgeries

### Risk factors for CRKP-BSI in pediatric patients

A multivariate logistic regression analysis was performed to identify independent risk factors for CRKP-BSI. The dependent variable was CRKP-BSI (yes vs. no), and the following independent variables, each coded as a binary variable, were entered into the model: age, underlying disease status, duration of antibiotic use ≥ 14 days, mechanical ventilation, surgical procedure, and length of hospital stay > 5 days. The results showed that mechanical ventilation, surgical procedure, and length of hospital stay > 5 days were independent risk factors for CRKP-BSI (all P < 0.05), as detailed in Table 3.

**Table 3:** Multivariate logistic regression analysis of risk factors for CRKP-BSI in pediatric patients

factor	β	SE	Wald χ2	OR(95%CI)	*P*
Mechanical ventilation	2.896	0.480	36.477	18.106(7.074,46.347)	0.001
Surgical procedure	1.116	0.559	3.992	3.052(1.021,9.120)	0.046
Hospital stay > 5 days	0.957	0.452	4.487	2.603(1.074,6.307)	0.034
Duration of antibiotic use ≥ 14 days	0.494	0.451	1.200	1.639(0.677,3.969)	0.273
constant	-2.016	0.610	10.935	-	0.001

“-”, the confidence interval of the constant term was not calculated

## Discussion

KP is one of the primary pathogens causing bloodstream infection (BSI); if not treated promptly, it can lead to septic shock, and is associated with high morbidity and mortality. Recent studies have reported that the detection rate of CRKP-BSI continues to rise, and it has emerged as the Enterobacteriaceae pathogen associated with the highest mortality in BSI. The World Health Organization (WHO) has noted that CRKP-BSI poses a serious threat to patients’ lives and has become a major public health problem [4].

In this study, the highest detection rates of CRKP-BSI were observed in the Intensive Care Unit (ICU) and the premature infant department. Patients in the ICU are more susceptible to infection because of reduced immune resistance, longer hospital stays, more invasive procedures, and greater antibiotic exposure [5]. These findings underscore the importance of strengthening hospital infection prevention and control measures, standardizing clinical procedures, and promoting the rational use of antimicrobial agents. Comparisons of departmental distribution revealed that the number of cases in the endocrinology department, infant department, infectious diseases department, gastroenterology department and nephrology-rheumatologyc department were too small to support valid statistical testing; therefore, between-group distribution differences for these departments could not be reliably assessed. However, significant between-group differences were found for the premature infant department, surgery department, and hematology-oncology department(all *P* < 0.05). This may be attributed to the fact that children in these departments are generally critically ill, with immature organ system development and compromised immune function, predisposing them to infections caused by multidrug-resistant organisms [6].

Analysis of clinical characteristics in the two groups also showed that risk factors for CRKP-BSI included age, underlying disease (prematurity, gastrointestinal disease, and malignancy), duration of antibiotic use, mechanical ventilation, and surgical history. Premature infants often present with multiple underlying conditions and immature immune, respiratory, and digestive systems, which increase their susceptibility to multidrug-resistant organism infections [7]. In-hospital antibiotic exposure, invasive procedures, and surgical interventions all increase the risk of infection, and infants and young children are more susceptible to CRKP infection [8]. CRKP can colonize the intestinal tract of children, and combined with immature immune function, endotracheal intubation and antibiotic use significantly increase the risk of CRKP bloodstream infection.

Multivariate logistic regression analysis revealed that mechanical ventilation, surgical procedures during hospitalization, and a hospital stay exceeding 5 days were independent risk factors for CRKP-BSI in pediatric patients. Mechanical ventilation is widely used in the resuscitation of premature infants and neonates, and related reports have shown that its associated bloodstream infection rate can reach 20.9% [9]. *Klebsiella pneumoniae*, *Acinetobacter baumannii*, and *Pseudomonas aeruginosa* are the main pathogens causing ventilator-associated bloodstream infections. Prophylactic antibiotic use during mechanical ventilation may lead to dysbiosis and the emergence of resistant isolates, increasing the risk of bloodstream infection with resistant organisms, a phenomenon also reported by Guang et al. [10]. Although duration of antibiotic use ≥ 14 days was not identified as an independent risk factor for CRKP-BSI in the present study, prolonged antibiotic exposure remains an important contributory factor. Long-term antimicrobial use can disrupt the homeostasis of the gut microbiota and promote colonization and infection with resistant organisms. Surgical procedures disrupt natural physical barriers such as skin and mucous membranes, and improper postoperative care may allow pathogens to invade the circulatory system, leading to bacteremia or sepsis [11]. The present study confirms that surgery is a significant risk factor for bloodstream infections. Long-term hospitalized patients often have underlying conditions (such as diabetes, malignancies, hepatic or renal insufficiency) and are more likely to experience malnutrition and prolonged bed rest [12], resulting in compromised immune function and reduced pathogen clearance capacity, which collectively elevate the risk of bloodstream infections. Moreover, hospital environments are high-risk areas for multidrug-resistant organisms (MDROs), and prolonged hospitalization increases the risk of MDRO colonization in the skin, respiratory tract, and other sites [13]. Colonizing bacteria serve as the primary source of bloodstream infections, and when host defense barriers are compromised, these bacteria can enter the bloodstream and cause infection [14]. Inappropriate or prolonged use of antimicrobial agents disrupts the balance of normal flora, promotes the proliferation of resistant bacteria, and leads to more difficult-to-treat resistant bloodstream infections.

This study has the following limitations: it is a single-center retrospective study, which may be subject to selection bias and information bias, and the data were derived from medical record systems, which may contain missing or incomplete information. This study used an unmatched case-control design and did not match for important confounders such as age and underlying disease; although multivariate regression was used for adjustment, residual confounding may remain. The sample size was limited and derived from a single institution, which may limit the generalizability and representativeness of the findings. In addition, molecular typing of isolates, resistance gene detection, and colonization screening were not performed, so the mechanisms of resistance and transmission could not be elucidated at the molecular epidemiological level.

In conclusion, mechanical ventilation, surgical procedures during hospitalization, and a hospital stay exceeding 5 days were identified as independent risk factors for CRKP-BSI in children. These findings underscore the importance of intensive monitoring and enhanced infection prevention and control measures in pediatric patients presenting with these high-risk factors, with early intervention to mitigate the risk of infection. Future multicenter, prospective studies with larger sample sizes, combined with molecular epidemiology and precision hospital infection surveillance, are needed to further validate these risk factors and improve prevention and control strategies, providing higher-level evidence-based support for the comprehensive prevention and treatment of bloodstream infections caused by drug-resistant organisms in children.

## Supplementary Information

Supplementary Material 1.

## Data Availability

The data generated and analyzed during the study are available from the corresponding author upon request.
