# Supplementary material for: Analysis of risk factors for carbapenem-resistant Klebsiella pneumoniae bloodstream infections in children: a case-control study
Source: BMC Microbiol. 2026 Jul 3;26:829. doi: 10.1186/s12866-026-05342-8 (PMC13602804; doi:10.1186/s12866-026-05342-8)
Supplement: Supplementary file 1 [file 12866_2026_5342_MOESM1_ESM.pdf]

耐药	科室	性别（男=1 女=2）	年龄	基础性疾	抗生素使用时间≥14d	抗生素使用≥3	免疫抑制剂使用情况	机械通气
00538768	东区血液	2	3y7m	急淋	否	是	使用	无
06010194	新生儿内	1	1m14d	早产	否	否	否	否
06016359	内科监护	1	2m8d	无	否	否	否	是
06018805	新生儿内	1	21d	无	否	是	无	否
06019877	新生儿重	2	13d	早产，先	否	是	无	是
06021489	新生儿内	1	18d	无	否	是	无	无
06022946	新生儿重	1	5d	早产，双	否	是	无	是
06025190	新生儿重	2	23d	早产	是		无	是
06032884	东区新生	2	4d	无	否	否	无	无
06032885	东区新生	1	8d	早产	否	是	无	是
06033127	东区新生	2	10d	早产，三	否	是	无	是
06035626	东区新生	2	5d	无	否	否	无	无
06036836	东区内科	1	1y7m	无	无	无	是	是
06036991	东区外监	1	6m25d	先心	否	是	否	是
06058605	东区新生	1	1d	先心	否	否	无	是
06065837	东区早产	1	12d	早产	否	否	无	是
06066535	东区血液	1	3y10m	急淋	否	否	有	无
06074541	东区早产	2	2m5d	早产	是	是	有	有
06075810	东区早产	2	16d	早产	是	是	无	有
06076729	东区新生	1	8d	无	否	是	无	无
06079119	东区早产	1	27d	早产	否	是	无	有
06080423	东区内科	2	14y	无	是	是	有	有
06083590	东区内科	1	1y10d	无	是	是	无	是
06088878	东区新生	1	14d	早产	否	是	否	是
06089530	东区早产	2	14d	早产	是	是	否	是
06096194	东区新生	2	12d	无	无	无	无	无
06097132	东区早产	1	26d	早产	是	是	无	有
06098115	东区早产	1	1d	早产	是	是	无	有
06099538	东区早产	1	13d	早产，大	否	是	无	有
06105548	东区早产	1	23d	早产	是	是	无	有
06115316	东区小儿	2	2m16d	无	无	无	无	无
06132925	东区早产	1	13d	早产	无	是	无	有
06134275	东区早产	1	22d	早产	是	是	无	有
06134582	东区早产	2	12d	早产	否	是	无	有
06136957	东区早产	2	4d	早产	是	是	有	有
06167516	东区新生	2	1d	消化道畸	是	是	无	有
06173442	东区早产	2	10d	早产双胎，小，巨型结肠	是	是	无	有
06174549	东区新生	1	1m13d	骶尾部畸	否	是	无	无
06175447	东区早产	1	8d	早产	否	是	无	有
06175448	东区早产	1	7d	无	否	是	无	有
06176099	东区新生	1	12d	早产，双	否	是	无	有

06177104	东区早产儿	1	4d	早产，双胎	是	是	有	有
06181576	东区早产儿	1	1d	早产	是	是	无	有
06181881	东区新生儿	2	15d	无	是	是	无	有
06186835	东区早产儿	2	1d	早产，先心	是	是	无	有
06188235	东区早产儿	2	15d	早产	是	是	无	有
06188413	东区早产儿	2	1d	早产	否	是	无	有
06193181	东区新生儿	1	3m16d	早产	否	否	无	无
06193185	东区早产儿	2	12d	早产，先心，小肠坏死	是	是	是	有
06193465	东区新生儿	1	8d	无	无	是	无	无
06195904	东区早产儿	1	19d	早产	是	是	有	有
06197278	东区小儿肝	2	6M1D	胆道闭锁术	无	无	无	无
06202083	东区早产儿	1	8d	早产，双胎	是	是	有	有
06205986	东区早产儿	1	11d	早产	是	是	有	有
06207376	东区早产儿	2	8d	早产	是	是	有	有
06210953	东区早产儿	1	1d	早产,先心	是	是	有	有
06211000	东区内科监	1	1y3m	无	是	是	无	无
06213411	东区早产儿	1	17d	早产	是	是	无	有
06214634	东区新生儿	1	8m	肠造瘘术后	否	是	无	无
06220916	东区早产儿	1	25d	早产	是	是	有	有
06221606	东区早产儿	1	15d	早产	是	是	有	有
06224669	东区早产儿	1	15d	早产	无	是	有	有
06229097	东区早产儿	1	3d	早产	是	是	有	有
06232706	东区早产儿	1	10d	早产	是	是	有	有
06237950	东区早产儿	2	1d	早产	否	是	是	有
06248790	东区外监胎	1	26d	先心	否	有	有	有
06249202	东区新生儿	1	25d	无	是	是	无	有
06249453	东区早产儿	2	5d	早产	是	是	有	有
06253161	东区外监胎	2	9m1d	先心	是	是	是	有
06259784	东区新生儿	1	28d	无	否	是	无	无
06263460	东区早产儿	2	2m4d	早产	是	是	是	有
06265892	东区内科监	2	6m21d	先心	否	是	无	有
06268356	东区新生儿	1	1m29d	早产	是	是	有	有
06271881	东区早产儿	1	9d	早产，双胎	否	是	无	有
06275415	东区新生儿	1	22d	早产，先天	否	否	否	否
06276071	东区新生儿	2	1m12d	早产	否	否	无	无
06278455	东区早产儿	2	3d	早产	是	是	有	有
06286483	东区内分泌	2	1y3m	无	否	是	无	无
06302278	东区早产儿	1	2d	早产，小	是	是	是	有
06303276	东区早产儿	1	1d	早产，先天	是	是	是	有
06306971	东区新生儿	2	11d	无	否	是	无	无
06308125	东区早产儿	1	25d	新生儿坏死	是	是	无	有
06311801	东区胸心外	1	1m25d	先心	是	是	是	有
06311884	东区外监胎	1	2d	先心	是	是	否	无
06317702	东区外监新	2	8d	早产	否	是	无	有
06319249	东区外监胎	1	1m2d	先心	否	是	是	有

06326671	东区新生丿	2	2m14d	先心	否	是	有	有
06358649	东区早产丿	2	18d	早产	否	是	有	有
06368187	东区新生丿	1	2m2d	早产，肠柞	是	是	是	有
06370574	东区早产丿	2	4m5d	早产，视网	是	是	有	有
06372897	东区早产丿	2	14d	早产	是	是	有	有
06379577	东区早产丿	2	7d	早产，先心	是	是	有	有
06379849	东区早产丿	1	17d	早产	是	是	有	有
06384094	东区新生丿	2	11d	无	无	是	无	有
06395371	东区普通纟	1	6m3d	先天性巨纟	是	是	无	无
06402319	东区新生丿	2	22d	早产,极低	是	是	有	有
06411233	东区新生丿	1	7d	无	无	是	无	无
06411714	东区早产丿	1	10d	早产，低亻	否	是	无	有
06413246	东区新生丿	1	1m28d	早产，低亻	是	是	是	有
06426879	东区外监肚	2	9d	早产，先心	是	是	有	有
06435231	东区新生丿	1	4d	早产，试饣	否	是	无	有
06442388	东区新生丿	2	26d	无	否	是	无	无
06444958	东区内科山	1	3y5m	无（溺水）	否	是	有	有
06478655	东区新生丿	1	2m6d	早产，肠柞	否	是	无	无
06484108	东区血液肚	1	25d	无	是	是	无	有
06488322	东区早产丿	2	8d	早产	否	否	无	无
06076058	东区小婴丿	2	4m7d	无	否	否	无	无
06087208	东区新生丿	2	20d	无	否	是	无	有
06106451	东区早产丿	2	28d	早产，极亻	是	是	是	有
06311206	东区血液肚	1	7m13d	无	是	是	是	有

动静脉插管	感染性休克	外科手术	住院时间	结局	白细胞	中性比例	淋巴细胞比	PLT
无	无	无	>20d	好转	0.28	10.7	89.3	79
否	否	否	13d	好转	2.24	69.1	28.9	170
否	否	否	12d	治愈	10.85	26.2	65.6	793
否	否	否	>20d	治愈	53.91	76	16.1	11
无	无	无	>20d	治愈	22.68	84.5	8.4	152
无	无	无	5d	治愈	9.61	40.8	46.3	241
无	无	无	5d	自动出院	4.46	39.8	45.5	20
无	无	无	17d	好转	10.05	45.6	37.6	20
无	无	无	1d	好转	7.19	64.2	20.9	53
无	无	无	8d	治愈	20.80	76.5	19.1	59
无	无	无	>10d	好转	8.08	70.4	21.0	4
无	无	无	3d	治愈	19.44	82.8	9.2	57
有	有	无	>30d	好转	11.01	88.0	11.3	173
有	无	有	》10	治愈	16.35	65.7	24.7	355
无	无	无	9d	自动出院	22.68	82.0	14.0	102
无	无	无	2d	死亡	15.74	41.4	48.9	4
有	无	无	18d	好转	0.17	17.6	64.7	106
无	无	无	20d	自动出院	9.49	50.3	34.6	8
无	无	无	》16d	好转	9.64	49	39.8	177
无	无	无	8d	治愈	18.95	77.8	13.7	54
无	无	无	8d	好转	9.6	37.3	41.3	185
有	有	无	》30d	好转	12.44	93.4	4.1	203
无	无	无	》20d	好转	21.73	73.6	24.8	126
无	无	无	3d	好转	21.15	78.8	13.0	36
无	无	无	14d	自动出院	4.09	54.0	40.1	186
无	无	无	2d	治愈	13.53	67.9	25.5	18
无	无	无	26d	好转	2.83	38.9	55.1	98
无	无	有	90d	好转	1.42	74.7	23.2	24
无	无	无	13d	自动出院	5.65	63.3	30.1	2
无	无	无	23d	好转	9.83	32.5	54.2	18
无	无	无	60d	好转	14.69	55.9	43.7	397
无	无	无	13d	好转	5.06	59.6	33.6	97
无	无	无	22d	好转	3	33.6	47.0	187
无	无	无	12d	自动出院	31.6	74.3	10.7	27
无	无	有	>30d	好转	10.19	79.1	17	33
有	无	有	>30d	自动出院	14.83	65.8	29.3	295
无	无	有	》60	好转	16.31	76.2	21.1	342
无	无	有	20d	好转	10.17	77.8	19	432
无	无	无	8d	好转	25.39	70.6	12.4	7
无	无	无	8d	自动出院	44.01	88.5	4.6	50
无	无	无	14d	自动出院	31.04	78.6	9.7	66

无	无	无	34d	好转	2.74	49.2	44.2	145
无	无	无	20d	好转	1.63	68.7	26.4	160
无	无	无	15d	自动出院	12.24	86.7	8.7	6
无	无	无	27d	好转	14.5	47.9	17.9	132
无	无	无	22d	好转	4.67	64.9	28.7	54
无	无	无	10d	好转	4.35	79.8	17.0	164
无	无	无	2d	好转	7.54	84.8	13.5	460
无	无	有		好转	1.76	68.1	26.7	49
无	无	无	10d	好转	5.43	39.6	51.2	4
无	无	无	>30d	好转	6.18	56.9	39.0	26
无	无	无	3d	好转	5.29	48	44	346
无	无	无	>30d	自动出院	2.70	73.3	21.9	200
无	无	无	>20d	好转	33.58	78.4	13.2	62
无	无	有	20d	好转	17.7	55.4	23.2	124
无	无	无	>30d	好转	1.15	18.3	73.9	92
无	无	无	21d	好转	23.27	96.3	2.9	423
无	无	有	>60d	自动出院	2.44	39.8	39.3	85
无	无	有	8d	好转	32.03	84.5	10.9	4
无	无	有	>30d	好转	2.29	50.6	43.2	253
无	无	无	15d	好转	37.22	78.9	15.3	122
无	无	无	10d	好转	6.36	68.4	26.3	249
无	无	无	>60d	好转	1.94	40.3	41.8	48
无	无	无	10d	好转	2.22	60.8	34.7	154
无	无	无	10d	好转	19.76	74.4	21.6	8
无	无	有	13d	痊愈	6.73	71.9	19.8	201
无	无	无	38d	自动出院	15.64	76.3	16.6	161
无	无	有	45d	好转	11.63	29.7	41.6	308
无	无	有	18d	痊愈	14.81	69.8	23.1	371
无	无	无	18d	自动出院	22.09	50.6	38.4	332
无	无	无	30d	好转	4.27	78.0	17.3	187
无	无	无	8d	自动出院	13.51	72.1	19.3	275
无	无	无	74d	好转	9.92	68.4	22.1	62
无	无	无	9d	自动出院	16.52	60.1	23.1	58
无	无	无	1d	好转	38.37	78.5	16.9	766
无	无	无	1d	痊愈	10.93	79.2	71.2	532
无	无	无	15d	好转	29.79	92.7	5.6	101
无	无	无	7d	好转	9.58	24.6	64.6	690
无	无	无	30d	好转	5.21	54.3	17.1	230
无	无	无	20d	好转	8.03	70.6	18.8	35
无	无	无	5d	好转	14.98	44.7	41.9	731
无	无	无	25d	痊愈	7.36	39.2	37.1	205
无	无	有	15d	痊愈	21.98	78.5	17.9	32
无	无	有	14d	痊愈	43.63	85.4	9.1	80
无	无	无	10d	好转	2.86	71.7	17.5	30
无	无	有	10d	治愈	10.6	67.30	23.6	560

无	无	无	5d	好转	12.06	67.2	25.3	214
无	无	有	7d	好转	17.33	75.7	12.2	10
无	无	有	64d	自动出院	20.22	80.4	15.9	8
无	无	有	21d	好转	9.9	66.8	26.7	7
无	无	无	28d	好转	12.38	58.9	20.4	99
无	无	有	27d	好转	7.95	90.4	7.9	3
无	无	无	17d	好转	13.29	64.3	25.6	4
无	无	无	10d	好转	14.35	81.4	14.3	77
无	无	有	14d	痊愈	2.32	7.3	90.1	178
无	无	无	30d	好转	2.89	50.6	40.5	68
无	无	无	5d	痊愈	28.86	54	31.8	100
无	无	无	10d	好转	16.18	76.8	17.6	18
无	无	有	30d	好转	3.33	71.5	26.1	294
无	无	有	26d	痊愈	24.28	76.8	15.0	125
无	无	无	10d	好转	4.01	65.1	24.2	199
无	无	无	8d	痊愈	6.20	78.2	18.7	118
无	无	无	8d	自动出院	4.26	61.9	1.37	29
无	无	无	10d	痊愈	34.36	94.2	4.3	120
无	无	有	22d	好转	8.25	87.0	11.9	68
无	无	无	1d	好转	10.00	71.2	21.4	71
无	无	无	2d	痊愈	10.25	37.3	53.5	526
无	无	无	7d	痊愈	3.20	66.7	28.8	218
无	无	无	35d	好转	16.31	92.8	6.5	199
无	无	无	40d	好转	11.21	65.2	29.9	631

CRP	PCT	肌酐	ALT
115.26			
59.41	13.41		
1.18		26.9	33
87.73	>100.0		27
46.9	79.79	75.7	18
68.51	7.56	17.9	22
5.52	1.12	79.8	427
30.77			
44.22	40.27		
60.23	29.56		20
51.31	3.37	32.7	16
55.57	84.47	90.7	6
55.27	0.302	10.2	22
39.39		34.6	20
80.26			18
《0.5	1.59	64.6	198
56.59	0.352		
26.69	68.61	57.8	83.1
<0.8	0.242	47.6	23.8
46.63	99.46	57.3	17.1
《0.8	0.314	114.1	10.2
60.12	51.8	35.5	42.4
	》100	39.8	541.8
《0.8	0.842	50.8	38.8
<0.8			
<0.8	33.28	62.8	29
39.87		89.5	545
12.29			
70.36		129.1	64.4
《0.8		52.2	29.2
97.56	0.595	14.6	72.8
1.55			
105.54		73.6	48.8
《0.8		41	22.5
65.94		28	27.6
40.81	3.97	25.4	86
12.82		15.5	50.5
91.79	1.86	32.7	43.1
11.27	6.77	77.3	126.6
71.05	44	88.7	33.5
32.26	42.57	88.9	23.3

4.71			
15.19		37.6	4.4
82.94	31.96	50.3	61.5
33.56			
<0.8			
37.03	0.138	26	17
<0.8	0.611		73.4
55.67		32	9.7
198.76	64.38	37.5	7
136.56			
2.77		32.7	151.4
23.98			
101.41	0.255	49.8	31.8
75.47		37.1	6
20		38.8	8.1
12.87	18.82	30.3	58.2
16.14			
116.85			
14		22.1	34.1
59.97		48.8	77.6
<0.8		140	20.8
122.02		31.6	8.8
《08		13.8	8.8
32.78			
72.44	3.9		
28.88	1.89		
12.71		34.6	329.3
8.46	0.127		20.7
9.24	0.188		
20.53		13.1	107.9
35.75	1.69	21.1	28.1
104.97	5.22	9.3	47.2
<0.5	1.421	27.2	26.3
39.5	2.04	83.3	61.6
41.83	10.57	34.0	
50.46	31.66	30.5	12.4
《0.5		13.3	7.5
172.09			
7.47			
<0.5	0.134	33.3	29.4
49.16	0.795	29.3	7.2
97.75		14.2	62.9
140.11	1.37		
21.25	12.03		2.2
17.78	0.092		

44.83	2.82		
170.13	40.15	78.9	16.2
170.38			57.2
149.70		22.8	5.7
118.45		15.8	14
29.39		89.9	18.2
1.06	3.9	26.7	19.2
100.63	3.18	34.4	20.7
39.12	8.64	19.7	27.5
2.24			
69.18	1.1	33.3	6
108.59	43.04	42.4	3.2
20.06	2.29	18.4	85
13.5	2.54		
17.41	0.117		
40.26	8.14		
232.2	3.75		82
66.92	36.99		
67.84	51.32		15.4
45.1		35	14
0.8	0.717	46.6	89.1
0.8	35.75	42.8	34.1
52.95		15.8	170
47.11	1.56	33.1	24.9

敏感

科室	性别（男=1 女=2）	年龄	基础性疾	抗生素使用时间≥14d	抗生素使用≥3	免疫抑制剂使用情况	机械通气	动静脉插
东区血液	1	5y3M	无	是	是	是	无	无
东区普通	1	3m	无	是	是	无	无	无
新生儿内	1	11d	早产	否	是	否	无	无
新生重症	1	17d	无	否	是	是	是	无
新生儿重	1	6d	无	否	是	无	无	无
消化内科	1	3m4d	无	无	无	无	无	无
血液肿瘤	1	7y	白血病	是	是	是	无	无
新生儿外	1	5d	无	无	无	无	无	无
东区新生	1	1m4d	无	是	是	有	无	无
东区新生	2	6d	先心	无	是	无	无	无
东区新生	1	7d	羊水混，	无	无	无	无	无
东区新生	2	2m1d	无	无	无	无	无	无
东区早产	1	14d	早产	无	有	无	无	无
东区早产	2	6d	早产	无	是	无	有	无
东区外监	2	4m13d	先心	是	是	是	有	无
东区新生	1	2m4d	无	无	有	无	无	无
东区新生	2	20d	无	无	无	无	无	无
东区新生	2	6d	无	无	是	无	无	无
东区新生	2	11d	无	无	有	无	无	无
东区外监	2	8d	先心	无	有	有	有	无
东区新生	1	1m1d	无	无	无	无	无	无
东区新生	1	3d	羊水浑浊	无	有	无	有	无
东区小婴	1	1m21d	出生窒息	无	无	无	无	无
东区小婴	1	1m2d	无	无	有	无	无	无
东区新生	2	1m1d	无	无	无	无	无	无
东区新生	1	1d	早产	无	有	无	无	无
东区新生	2	23d	无	无	无	无	无	无
东区新生	2	10d	早产	无	有	无	有	无
东区早产	2	10d	早产	无	有	无	有	无
东区消化	1	2y9m	无	无	无	无	无	无
东区新生	1	9d	早产，窒	无	有	有	有	无
东区早产	1	11d	早产，先	无	有	有	有	无
东区早产	2	11d	早产，呼	无	有	有	有	无
东区新生	1	6d	试管，足	无	有	无	无	无
东区普通	2	6m	胆道闭锁	有	有	无	无	无
东区新生	1	13d	无	无	无	无	无	无
东区新生	1	7d	无	无	无	无	无	无
东区早产	1	1d	早产	无	无	无	有	无
东区新生	1	3d	无	无	有	无	无	无
东区新生	1	19d	无	无	无	无	无	无
东区早产	1	6d	早产，双	有	有	无	有	无
东区新生	2	11d	缺氧	无	无	无	无	无

东区新生	1	4d	无	无	无	无	无	无
东区新生	1	4d	消化道畸	无	无	无	无	无
东区新生	1	2m1d	喂养困难	有	有	无	无	无
东区血液	2	3y3m	白血病再	无	有	无	无	无
东区早产	1	1d	早产	无	无	有	有	无
东区新生	1	1m17d	无	无	有	无	无	无
东区新生	1	12d	先天性巨	无	无	无	无	无
东区早产	1	17d	21三体，	无	有	有	有	无
东区新生	2	4d	无	无	有	有	无	无
东区新生	1	4d	无	无	有	无	无	无
东区早产	1	9d	双胎，早	无	无	无	无	无
东区早产	1	5d	早产	有	有	有	有	无
东区早产	2	4d	早产，呼	无	有	有	有	无
东区早产	2	1d	早产，低	有	有	有	有	无
东区肾脏	2	5m10d	无	有	有	有	无	无
东区新生	1	10d	无	无	无	无	无	无
东区新生	1	5d	早产，呼	无	有	有	有	无
东区新生	1	3d	无	无	有	无	无	无
东区早产	2	1d	早产呼吸	有	有	有	有	无
东区新生	1	5d	无	无	有	有	无	无
东区早产	1	14d	双胎，早	有	有	有	有	无
东区小儿	2	3m7d	胆道闭锁	无	无	无	无	无
东区小儿	1	2y7m	腹胀1年，	有	有	无	无	无
东区内科	2	1y10m	曾，腹腔	无	无	无	无	无
东区新生	2	7d	无	无	无	无	无	无
东区新生	1	11d	无	无	有	无	无	无
东区小儿	2	1y7m	胆道闭锁	无	无	无	无	无
东区小婴	1	3m25d	无	无	无	无	无	无
东区血液	1	10y	确诊急性	有	有	有	无	无
东区小儿	1	3m5d	胆道闭锁	无	无	无	无	无
东区血液	1	2y6m	[确诊B淋	有	有	有	无	无
东区血液	1	8y	确诊急性	无	有	有	无	无
东区新生	1	1m14d	无	无	无	无	无	无
东区外监	2	7y	确诊急性	无	有	有	无	无
东区消化	2	3m1d	胆管闭锁	无	无	无	无	无
东区血液	2	10y	再障	有	有	有	无	无
东区小儿	2	4m5d	胆道闭锁	无	无	无	无	无
东区血液	1	12y	确诊急性	有	有	有	无	无
东区新生	1	2m1d	无	无	无	无	无	无
东区血液	2	5m3d	确诊急性	有	有	有	无	无
东区血液	1	12y	确诊急性	有	有	有	无	无
东区外监	1	4m15d	先天性小	无	有	无	无	无
东区血液	1	2y6m	确诊B淋巴	无	有	有	无	无
东区血液	1	12y	确诊急性	无	有	有	无	无
东区血液	1	3y3m	确诊高级	有	有	有	无	无
东区内科	2	2m28d	先心	无	无	无	无	无
东区血液	1	8y	[诊断成熟	有	有	有	无	无

东区神经	1	2y1m	早产，先	有	有	有	有	无
东区急诊	1	9m20d	无	无	无	无	无	无
东区新生	2	4d	无	有	有	有	有	无
东区血液	1	13d	[确诊急性	有	有	有	无	无
东区新生	1	4d	先天肛门	无	有	无	无	无
东区早产	1	26d	早产，呼	有	有	有	有	无
东区小儿	2	1y2m	先天胆道	无	无	无	无	无
东区血液	1	10y	确诊急性	有	有	有	无	无
东区新生	1	1d	早产	有	有	有	有	无
东区内科	1	3m10d	无	有	有	有	有	无
东区新生	1	3m2d	回肠造瘘	有	有	无	无	无
东区新生	2	11d	早产生后	有	有	有	有	无
东区血液	2	2y10m	确诊神经	无	有	有	无	无
普通外科	1	2m11d	胆道闭锁	无	无	无	无	无
东区新生	2	1m4d	无	无	有	有	无	无
东区新生	1	1d	早产儿、	无	有	无	无	无
东区血液	1	1y7m	无	无	有	无	无	无
东区内科	2	1y10m	发现血细胞异常1月		有	无	无	无
东区内科	2	3m12d	遗传代谢	有	有	有	有	无
东区血液	2	12y	确诊T淋巴	有	有	有	无	无
东区血液	2	5m26d	急性淋巴	无	无	无	无	无
东区小婴	1	4m19d	胆管闭锁	无	无	无	无	无
东区早产	1	4d	无	无	有	有	无	无
东区血液	1	6y1m	确诊髓母	无	无	无	无	无
普通外科	1	5m2d	胆道闭锁	无	有	无	无	无
东区血液	1	9y	确诊急性	无	无	无	无	吴
东区血液	1	2y3m	确诊急性	有	有	有	无	无
普通外科	2	3m11d	胆道闭锁	无	无	无	无	无
普通外科	1	14d	左肾上方	无	有	有	无	无
普通外科	1	2y3m	左侧肾母	无	有	有	无	无
东区感染	2	4m1d	无	无	无	无	无	无
东区消化	2	4m9d	无	无	无	无	无	无
东区血液	2	10y	诊断急性	有	有	有	无	无
东区肾脏	2	3m15d	无	无	无	无	无	无
东区血液	2	15y	确诊急性	有	有	有	无	无
东区早产	1	30d	早产，呼	有	有	有	有	无

感染性休	外科手术	住院时间	结局	白细胞	中性比例	淋巴细胞	PLT	CRP
无	无	14d	好转	0.34	8.8	85.3	13	7.77
无	是	51d	痊愈	18.07	72.6	17.2	57	10.36
无	无	11d	好转	6.12	56.4	35.1	10	86.91
无	无	3d	好转	6.66	48.8	33.5	53	87.78
无	无	3d	痊愈	11.05	60.3	19.6	8	14.98
无	无	1d	自动出院	24.12	25.1	68.0	174	14.54
无	无	53d	好转	0.73	1.3	97.3	6	<0.5
无	无	1d	自动出院	11.06	77.5	13.9	198	56.41
无	无	>30d	痊愈	22.85	81.0	14.0	3	120.25
无	无	6d	痊愈	39.13	87.6	9.1	31	27.02
无	无	1d	痊愈	10.23	78.7	11.0	90	48.33
无	无	1d	好转	20.33	55.9	34.3	306	55.79
无	无	7d	好转	16.28	58.4	29.4	55	39.84
无	无	3d	痊愈	22.95	82.0	10.5	15	65.59
无	有	30d	痊愈	9.96	50.9	40.9	252	7.42
无	无	9d	痊愈	37.29	86.6	10.1	35	87.65
无	无	1d	痊愈	13.49	82	9.5	53	0.5
无	无	6d	好转	17.57	86.4	6.9	103	18.17
无	无	3d	痊愈	4.75	82.7	11.6	39	1.75
无	是	11d	自动出院	37.74	88.7	6.1	121	63.45
无	无	1d	痊愈	12.29	34.4	50	369	<0.5
无	无	3d	痊愈	8.11	75.4	16	42	69.9
无	无	1d	自动出院	6.06	25.2	56.6	204	7.14
无	无	9d	痊愈	6.02	23.5	62	576	<0.8
无	无	1d	痊愈	8.61	69.7	25.2	166	29.59
无	无	4d	放弃，自	3.59	47.7	44	1	59.82
无	无	1d	痊愈	9.22	79.8	10.8	100	53.25
无	无	10d	痊愈	3.98	43.6	33.2	197	7.39
无	无	10d	痊愈	30.68	93.1	2.6	357	0.8
无	无	1d	痊愈	6.63	20.2	70.4	179	0.8
无	无	9d	痊愈	41.66	76.5	12.3	53	0.8
无	无	11d	痊愈	7.67	82.8	11.9	361	0.8
无	无	11d	好转	10.89	84.8	6.9	114	<0.8
无	无	6d	治愈	13.31	56.1	30.4	100	142.83
无	无	18d	好转	9.26	51.3	44.9	213	68.19
无	无	2d	治愈	33.15	88	9.5	3	<0.8
无	无	1d	治愈	8.51	32.4	47	129	7.32
无	无	26d	好转	4.90	76.7	18.2	228	<0.8
无	无	5d	治愈	22.39	91.9	6.7	27	125.53
无	无	1d	治愈	13.39	69	21	266	14.61
无	无	60d	好转	3.59	5	91.1	239	17.47
无	无	2d	治愈	15.99	74.6	18.3	363	52.19

无	无	10d	治愈	6.56	34.7	59.8	4	44.30
无	无	1d	好转	12.05	64.5	26.2	432	16.28
无	无	40d	好转	5.41	69.5	26.2	56	40.09
无	无	13d	好转	0.23	69.7	21.7	170	198.23
无	无	22d	好转	8.34	36	46.8	356	38.09
无	无	5d	治愈	8.90	69.4	25.5	126	52.21
无	无	1d	好转	8.94	43.1	49.7	554	<0.8
无	无	3d	自送出院	14.21	74.4	15.5	2	83.05
无	无	1d	好转	17.35	81.5	14.9	14	<0.8
无	无	3d	好转	3.41	58.3	35.8	108	10.34
无	无	1d	治愈	4.92	37	34.1	79	100.18
无	无	45d	好转	3.71	30.7	65.2	255	9.26
无	无	6d	好转	13.78	87.3	7.2	8	63.97
无	无	24d	死亡	16.07	53.5	24.2	3	78.86
无	无	17d	自送出院	9.60	36	56.5	189	0.5
无	无	1d	治愈	26.91	83.3	8	19	161.02
无	无	5d	好转	10.08	64.7	21.2	12	36.06
无	无	3d	治愈	0.59	67.8	25.4	101	74.62
无	无	15d	治愈	1.5	14.6	82.7	248	<0.8
无	无	4d	好转	18.9	88.4	6.4	234	44.38
无	无	14d	好转	15.38	89.8	6.7	37	27.16
无	无	1d	好转	17	66.7	28.6	450	96.6
无	无	30d	好转	13.24	81.1	13.4	442	29.02
有	无	1d	自动出院	44.29	76.2	20.3	268	16.75
无	无	2d	治愈	7.26	76	19	255	27.81
无	无	3d	治愈	12.41	46	41.8	126	10.77
无	无	1d	好转	24.70	70	26.1	315	216.30
无	无	1d	好转	17.97	71.4	22.3	602	32.47
无	无	15d	好转	1.23	2.5	95.9	20	
无	无	1d	好转	10.41	57.1	38.6	566	166.05
无	无	14d	好转	11.17	88.9	4.3	91	96.47
无	无	11d	好转	0.51	5.9	94.1	61	<0.8
无	无	1d	治愈	13.09	80.8	15.4	565	152.31
无	无	9d	自动出院	11.83	94	4.6	8	93.16
无	无	1d	好转	9.89	46.8	42.2	102	135.35
无	无	72d	好转	0.47	27.7	70.2	47	74.21
无	无	1d	治愈	8.56	83.6	11.2	202	73.06
无	无	19d	好转	0.17	5.9	94.1	15	95
无	无	1d	好转	4.21	17.1	71.5	398	<0.5
无	无	14d	好转	0.15	0.0	100	23	
无	无	14d	好转	0.84	3.6	94	22	
无	无	7d	治愈	12.63	79.5	17.4	477	63.99
无	无	12d	好转	0.12	8.4	83.3	27	
无	无	12d	好转	0.22	13.6	86.4	31	15.98
无	无	35d	好转	0.18	0	22.2	33	
无	无	1d	好转	97.42	78.9	16.1	117	32.34
无	无	22d	好转	14.91	94.3	5.5	116	1.26

无	有	33d	治愈	4.31	51.1	45.7	179	25.22
无	无	1d	好转	35.31	87	8.6	46	210.4
无	无	16d	好转	10.81	39.8	46.9	556	<0.5
无	无	33d	好转	1.35	8.9	88.9	138	80.92
无	有	5d	好转	6.15	33	48.6	341	<0.5
无	无	26d	好转	8.79	49.1	31.6	178	6.24
无	无	1d	好转	29.19	43.1	49.5	660	7.22
无	无	15d	好转	0.81	30.8	63	42	<0.8
无	有	120d	治愈	15.46	85.9	7.3	147	88.7
无	无	15d	好转	4.30	64	29.5	198	26.39
无	无	13d	治愈	3.02	67.9	28.5	194	25.05
无	无	13d	治愈	46.60	89.7	8.2	34	110.9
无	无	12d	好转	0.35	19.9	74.3	17	52.28
无	无	1d	好转	11.12	61.7	33.4	380	22.19
无	无	5d	治愈	13.74	66	29.7	39	126.43
无	无	7d	自动出院	13.74	36.6	45.9	472	3.8
无	无	11d	好转	0.15	40	60	38	2.63
无	无	1d	自动出院	8.08	53.2	39.9	35	156.36
无	无	22d	自动出院	9.09	85.1	9.5	398	14.9
无	无	13d	好转	1.03	87.4	12.6	48	0.74
无	无	1d	自动出院	562.78	18.6	41.8	28	2.35
无	无	1d	好转	17.43	12.7	79.5	240	2.26
无	无	4d	好转	19.08	84.8	7.5	90	76.53
无	无	2d	好转	2.85	85.1	7.4	239	<0.5
无	无	11d	好转	18.29	35.1	54.2	328	0.75
无	无	2d	好转	3.55	3.0	96.1	16	
无	无	14d	好转	0.05			51	15.52
无	无	1d	好转	8.37	71.7	25.5	230	66.12
无	有	10d	好转	3.31	75	19.9	125	64.77
无	无	5d	好转	3.48	74.7	23	176	1.81
无	无	2d	好转	13.78	33.3	60.9	215	85.47
无	无	1d	好转	25.76	75.1	22.2	602	41.42
无	无	21d	好转	0.29	6.9	93.1	30	0.59
无	无	1d	好转	12.88	71.4	19.2	289	117.62
无	无	16d	好转	0.15	0	100	19	6.37
无	无	30d	好转	8.27	56.1	34.6	12	62.93

PCT	肌酐	ALT
	29.7	129.2
0.55		477.9
24.91	68.1	25
2.33	76.7	25
4.9	44.5	11
2.42	16.5	176
0.279		
	120.9	8
13.33	21	30
29.69	54.5	16
77.84	46.1	14
0.309	16.4	24
53.5	45.2	31
100	69.2	49
3.51	45.7	28
76.32	30.7	114.5
100	45.9	30
100	44.3	12
100	32.6	39
64.88	46.4	128
0.082	25.8	33
72	86.6	21.1
11.92	34.3	45.9
0.063	42.9	44.7
2	41	45.6
1.17	117.2	18.6
100	73.7	52.5
0.203	147.2	15.2
44.13	67.9	17.1
0.253	25.1	29
41.39	83.3	44.5
	56	27
73.49	49	28.5
3.14	58.9	23.1
0.151	13.5	60.8
84.54	81.1	39.7
>100.0	57.1	9.9
	92.7	27
>100.0	149.3	131.9
6.11	48.7	87.3
	23.5	59.1
1.11	19.6	18.1

10.06		
>100.0	165.7	23.4
>100.0	27.6	127.7
0.34	15.5	12.5
42.67	34.9	320.4
0.147	51.5	29.4
11.63	55.8	31.6
17.4	95.3	20.1
5.42	60.9	23.6
21.72		
20.17		
0.162	37.2	15
>100.0	108.6	44.6
	63.5	30
25.68	72.6	25
0.147		
	37.7	5.9
9.27	13.3	183.1
	13.26	50.6
	66.3	584.4
0.704	27.9	10.4
5.73	13	56.2
0.471	25.3	13.9
2.86		
	29.5	142.3
4.52		
	23.2	36
25.92		
18.82	27.8	85.8
0.392		
0.083	23.4	42.7
0.224		
0.153		
21.59	105.3	36.1
35.31		

11.29	73.5	19.5
0.154	52.1	21.6
	28.7	11.2
1.1		
2.05	37.7	48.5
74.11	13.3	54.3
0.47		
18.49		
	24.6	437
>100.0	35.5	527
16.44	13.26	149
10.55	17.9	22
0.389		
0.204		
0.269	13.6	
	9.4	149.9
	87	12
46.57		
25.92		
6.08	51.6	43
0.245		13
6.92	10.9	82.1
2.33	23	18
5.85	25.7	25.3
2.45	13.3	38
5.92	19	188
0.493	25.7	62.7
0.343	21.4	17.5
0.204	32.9	219
36.11	32.8	11
